# Applying an equity lens to social prescribing

**DOI:** 10.1093/pubmed/fdae105

**Published:** 2024-06-25

**Authors:** Koser Khan, Stephanie Tierney, Gwilym Owen

**Affiliations:** National Institute for Health Research Applied Research Collaboration (NIHR ARC-NWC), Division of Health Research, Faculty of Health and Medicine, Lancaster University, Lancaster LA1 4AT, UK; Nuffield Department of Primary Care Health Sciences, University of Oxford, Woodstock Road, Oxford, OX2 6HT, UK; Department of Public Health Policy and Systems, University of Liverpool, L69 3GF, UK

**Keywords:** health inequalities, measurement, public health, social determinants, social prescribing

## Abstract

**Background:**

Social prescribing is often described as an intervention that can help reduce health inequalities yet there is little evidence exploring this. This study aimed to assess the feasibility of accessing and analysing social prescribing (SP) service user data to demonstrate the impact of SP on health inequalities.

**Methods:**

The sample size consisted of records for 276 individuals in Site 1 and 1644 in Site 2. Descriptive analyses were performed to assess the characteristics of people accessing SP, the consistency of data collected and the missingness across both sites.

**Results:**

Both sites collected basic demographic data (age gender, ethnicity and deprivation). However, data collection was inconsistent; issues included poor recording of ethnicity in Site 2, and for both sites, referral source data and health and well-being outcome measures were missing. There was limited data on the wider determinants of health. These data gaps mean that impacts on health inequalities could not be fully explored.

**Conclusions:**

It is essential that SP data collection includes information on user demographics and the wider determinants of health in line with PROGRESS Plus factors. Considering equity around who is accessing SP, how they access it and the outcomes is essential to evidencing how SP affects health inequalities and ensuring equitable service delivery.

## Introduction

Social prescribing (SP) is a mechanism for enabling general practitioners (GPs) and other health and community-based staff to connect people to local, non-clinical services and support.^1^ This is often through referral to a SP link worker, who is employed to support people with social (e.g. loneliness), economic (e.g. unemployment) and environmental (e.g. housing) problems affecting their health and well-being. SP is part of NHS England’s Long-Term Plan to incorporate person-centred care into the health and care system.^2^

SP is widely presented as an intervention to address wider social determinants of health (e.g. employment, housing, social isolation).^3–7^ Approaches within SP to target health inequalities include preventing or reducing progression of chronic disease via physical activity or healthy eating interventions, as well as linking individuals to financial, housing, leisure or social support.^8–10^ However, recent critical discourses suggest that SP may exacerbate health inequalities due to its focus on individual level change.^11–14^

Systematic reviews on SP have focused more broadly on health outcomes, health services use and general effectiveness/implementation.^9^^,^^15–17^ An equity lens is often not applied in the interpretation of findings. For example, where outcomes are considered in studies, researchers do not necessarily explore variations in outcomes or whether needs were met across different population groups.^18^^,^^19^ Therefore, research demonstrating impact on health inequalities has been limited.^12^

Initial scoping of SP services across North West England (*n* = 12) when planning this study identified that a number of providers collect a large amount of data on people accessing their service. This paper presents findings from a study involving two of these organizations. It considered challenges associated with using existing data held by SP organizations to demonstrate the reach and impact of SP on health inequalities.

## Methods

Two SP organizations (Sites 1 and 2), both commissioned through primary care networks (PCNs), agreed to participate. Site 1 is delivered through a local authority and Site 2 by a voluntary sector organization. Information sharing agreements were set up with organizations to enable access to existing data. Data extraction was anonymous and contained no personal data. Data were extracted by each organization from April 2020 to April 2021 for any individual in each organization’s SP case management system. This information was shared with a designated researcher.

Sample size included records for 276 individuals in Site 1 and 1664 in Site 2. Site 2 was more established and had been running for 5 years. Site 1 had been in operation for 12 months at the time of data collection. Variables of interest included age, gender, ethnicity, deprivation and any other socioeconomic information recorded such as employment and housing status. We also planned to explore referral source to review any variations in groups being referred (e.g. in terms of age or ethnicity) by professionals, duration cases remained opened, those disengaging with the service, recorded health and well-being outcome measures and variations in outcomes based on demographic factors.

The key explanatory variables in the analysis were age, gender, ethnicity and area level deprivation. Ethnicity was broken down into four groups: White, Pakistani, Indian and mixed/other. These categories were chosen due to the nature of the study areas, which had sizeable White, Pakistani and Indian populations but very small numbers of individuals from other ethnic groups.

Area level deprivation was taken from the 2019 Indices of Multiple Deprivation produced by the Ministry of Housing, Communities and Local Government at Lower-layer Super Output Area (LSOA) level.^20^ We used postcodes to match each individual to their LSOA. LSOAs are small geographical areas with an average population of 1500.^21^

Data analysis was exploratory. To understand who was accessing SP services, we estimated the percent of people in each sociodemographic group using SP and compared this to the general population for the area. For the general population, we used the 2020 mid-year population estimates^22^ for age and gender and the 2011 census^23^ for ethnicity. For deprivation, we split each site into quintiles of deprivation in that area. To explore how people were accessing SP, we looked at data on who referred each individual. To assess the potential impact of SP on outcomes, and any variations amongst groups, we intended to use data recorded from two outcome measures being used in the study sites. Site 1 used the Outcome Home Star and ONS4 wellbeing measures, while Site 2 only used the ONS4 well-being measure.^24^^,^^25^

## Results

We found data sharing agreements were relatively straight forward to set up and access the data. We found that each site had information collection processes that link workers were expected to complete. This included inputting demographic details, referral source and need, actions undertaken and outcome measures into their case management systems.

Table 1 provides sociodemographic data for each site. Service user data were limited to age, gender, ethnicity and deprivation. Other information, such as employment or housing, was not recorded by either site. Actions being undertaken by link workers and duration of case length were only recorded in Site 1. Hence, we were unable to analyse differences in case lengths across groups and case duration in Site 2. Ethnicity data for Site 2 were of poor quality and are listed as Missing in Table 1.

**Table 1:** SP statistics by sites and demographics

	Site 1 (Sample size *n* = 276)	Site 2 (Sample size *n* = 1664)
	Percentage of adults accessing SP	Percentage of adults accessing SP
Age		
19–30	12%	13%
31–40	12%	16%
41–50	17%	14%
51–60	19%	20%
61–70	12%	13%
71–80	11%	11%
81+	17%	12%
Ethnicity		
White	81%	Missing
Pakistani	9%	Missing
Indian	4%	Missing
Other	6%	Missing
Gender		
Men	40%	38%
Women	60%	62%
Deprivation		
Most deprived 20%	33%	35%
Second most deprived 20%	27%	26%
Middle 20%	16%	19%
Second least deprived 20%	16%	12%
Least deprived 20%	8%	8%

There were inconsistencies in information recorded around referral sources. In Site 1, most referrals were recorded as PCN referrals, so came from GP practices. In Site 2, data were missing in approximately half of cases, with the Clinical Commissioning Group listed as the main referral source. Thus, it was not possible to demonstrate which professional or community roles were referring into the services to explore any variations. We also found that both sites did not have data fields that captured disengagement with the service; hence, it was not possible to extract information on those who did not engage with SP after referral.

There was a large proportion of missing data on outcome measures related to well-being. In Site 1, 7% of service users completed the ONS4 well-being measure and 17% had completed the well-being star. In Site 2, 13% completed the ONS4 measure. Therefore, it was not possible to assess overall impact on well-being or if some groups had poorer or better outcomes than others.

### Discussion: Main findings

We investigated if routinely collected data held by SP organizations would enable us to explore equity issues around the use and benefits of SP services. We found both sites had data collection processes in place and data were accessible; however, significant data gaps existed, preventing us from adequately exploring the impact of SP in relation to equity. Information that could help better understand access and outcome inequities, such as ethnicity, and data on the wider determinants of health, as well as pre and post health and well-being outcome measures, were not routinely collected by the two sites involved.

### What we already know about this topic and what the study adds

Whilst this study reflects the need for greater consistency in SP data collection,^17^^,^^18^^,^^26^ more specifically it emphasizes the lack of consideration of health inequalities as part of routine data collection to monitor service reach and to support the targeting of provision.^12^^,^^27^^,^^28^

There is no standardized approach to collecting data and measuring outcomes for SP; although a minimum dataset has been outlined by NHS England and an outcome framework exists, these are not mandatory requirements.^29–31^ The onus to collect data and what to collect is left to individual services.

### Implications for research and practice

We propose that PROGRESS-Plus should be considered when developing data collection systems for SP and evaluating impact. They provide measures of inequalities in outcomes between groups and populations across the following: Place of residence, Race, Ethnicity, Cultural background, Occupation, Gender, Sex, Religion, Education, Social capital, Socioeconomic status as well as additional factors (e.g. disability and age).^27^ It is important for service commissioners and providers to understand the profiles of their local populations to identify underserved groups and those at risk, to effectively deliver interventions aimed at reducing health inequalities.^32^

We recognize that there may be challenges in collecting such data. However, several data collection tools are available specifically for SP, such as Elemental, Joy, Social Rx, which not only support better data collection but also help demonstrate outcomes. It is important that link workers are provided with appropriate training to support data collection and that they are given sufficient time for data entry.

Equity in relation to SP needs to study who is not and who is accessing SP, how they access it, reported outcomes and variation amongst groups. Intersectionality should also be considered. This will ultimately support the evidence base for SP, particularly in relation to health inequalities, and can inform actions taken by commissioners and providers to support more equitable service delivery and access.

Whilst this paper focused on existing data collected by SP organizations, the authors note that other important aspects of equity, such as how SP is being delivered and the availability of local resources that support SP activities, also need to be investigated to provide greater insights into equity. For example, we know that a key element of SP remains focused on individual behaviour change^33^^,^^34^ yet evidence suggests that interventions focusing on individual changes in knowledge, motivation and behaviour can increase health inequalities.^35^^,^^36^

## Limitations

Data came from two sites in North West England, so findings may not be generalizable to other parts of the country. There was significant missing data, which highlights the need for better recording of routine information that incorporates the wider determinants of health to be useful in assessing SP. We also recognize that inequalities associated with access to SP can be shaped by variation in the availability of non-clinical services and support in an area, and in how the link worker role is being implemented in a setting (e.g. how they receive referrals, whether they take a proactive approach to SP, how long they can spend supporting patients).

## Notes

**Koser Khan**, Senior Research Associate

**Stephanie Tierney**, Associate Professor

**Gwilym Owen**, Research Associate
