# Correction to: Applying an equity lens to social prescribing

**DOI:** 10.1093/pubmed/fdae182

**Published:** 2024-09-12

This is a correction to: Koser Khan, Stephanie Tierney, Gwilym Owen, Applying an equity lens to social prescribing, *Journal of Public Health*, Volume 46, Issue 3, September 2024, Pages 458–462, https://doi.org/10.1093/pubmed/fdae105

In the originally published version of the manuscript, funding information was unfortunately omitted.

The following has now been added to the article:

“**Funding**

This research is supported by the National Institute for Health Research Applied Research Collaboration North West Coast (ARC NWC). The views expressed in this publication are those of the author(s) and not necessarily those of the National Institute for Health Research or the Department of Health and Social Care.”

